# Clinical assessment and characterization of a dual‐tube kilovoltage X‐ray localization system in the radiotherapy treatment room

**DOI:** 10.1120/jacmp.v9i1.2318

**Published:** 2008-01-13

**Authors:** Sung‐Woo Lee, Jian‐Yue Jin, Huaiqun Guan, Flavious Martin, Jae Ho Kim, Fang‐Fang Yin

**Affiliations:** ^1^ Radiation Oncology St. Anne's Hospital Fall River Massachusetts; ^2^ Department of Radiation Oncology Henry Ford Health System Detroit Michigan; ^3^ Department of Radiation Oncology Duke University Medical Center North Carolina U.S.A.

## Abstract

Although flat‐panel kilovoltage X‐ray imaging devices have been well tested for clinical use in diagnostic radiology, their use as a part of an image‐guided radiation therapy (IGRT) system in a treatment room is new and requires systematic assessment.

We used the Novalis IGRT system (BrainLAB, Feldkirchen, Germany) for the present study. The system consists of two floor‐mounted kilovoltage X‐ray tubes projecting obliquely onto two flat‐panel detectors mounted on the ceiling. The system can automatically fuse two‐dimensional localization images with three‐dimensional simulation computed tomography images to provide positioning guidance. We evaluated these system parameters:

- Overall performance of the IGRT system, including isocenter correlation between the IGRT system and the linear accelerator (LINAC)
- Image quality of the system
- Exposure received by patients for a pair of images
- Linearity, uniformity, and repeatability of the system

The Novalis system uses a daily isocenter calibration procedure to ensure consistency of isocenters between the IGRT and the LINAC systems. The localization accuracy was about 1 mm. We measured the relative modulation transfer function (RMTF) to quantify the spatial resolution of the imaging device, with $f_{50}$ being 0.7 – 0.9 line pairs per millimeter. The maximal exposure of an image was 95 mR. We derived an empirical relationship between the exposure and the X‐ray technical settings. The other parameters of the system were quantitatively measured and generally met the requirements.

The IGRT system is safe and reliable for clinical use as a target localization device. The measured data can be used as a benchmark for a quality assurance procedure.

PACS number: 87.56‐v

## I. INTRODUCTION

Image‐guided radiation therapy (IGRT) plays an important role in intensity‐modulated radiotherapy (IMRT) and linear accelerator (LINAC)–based stereotactic radiosurgery because of the requirement by those techniques for precise localization of the target. Various IGRT systems have been developed and are currently used in clinics.^(^ ^1^ ^–^ ^8^ ^)^ These include ultrasonography‐based localization systems,^(^ ^1^ ^–^ ^2^ ^)^ flat‐panel two‐dimensional (2D) kilovoltage or megavoltage X‐ray imaging systems,^(^ ^3^ ^–^ ^4^ ^)^ and three‐dimensional (3D) kilovoltage or megavoltage IGRT systems such as cone‐beam computed tomography (CT).^(^ ^5^ ^–^ ^8^ ^)^ Although kilovoltage X‐ray units in combination with flat‐panel detectors have been well tested for clinical use in diagnostic radiology, their application as an image guidance system in a treatment room is relatively new. The purpose, frequency of use for patients, system configuration, and component properties of such units in an IGRT setting could be substantially different from those in the diagnostic setting.

The Novalis ExacTrac IGRT system (ExacTrac 3.5: BrainLAB, Feldkirchen, Germany) is a kilovoltage, X‐ray–based, 2D‐to‐3D, image‐fusion‐guided target localization device dedicated for cranial and extracranial stereotactic radiosurgery.^(^ ^4^ ^,^ ^9^ ^–^ ^11^ ^)^ As compared with diagnostic kilovoltage X‐ray or other kilovoltage IGRT systems, the Novalis system's unique configuration includes

- two X‐ray tubes and corresponding detector panels in fixed positions;
- projections from the two X‐ray tubes in oblique directions that are non‐orthogonal to one another; and
- source isocenter and source–detector distances that are relatively large (2.24 m and 3.62 m, respectively).

In X‐ray imaging applications, the basic imaging characteristics of the Novalis IGRT system—which include image quality; radiation exposure for each image; and system linearity, repeatability and uniformity—are important parameters for the machine's optimal and safe use in daily clinical practice. On the other hand, in target localization applications, the abilities of the Novalis to fuse localization and simulation images, to accurately readjust patient position according to the fusion result, and to retain coincidence of the isocenters between the IGRT and the LINAC systems are three essential elements in assuring satisfactory performance. The imaging characteristics of the X‐ray device can also affect the results of image fusion and hence the ultimate performance of the IGRT system. The purpose of the present study was to systematically evaluate these parameters in the Novalis system—specifically:

- overall performance of the IGRT system, including isocenter correlation between the IGRT system and the LINAC;
- image quality of the system;
- exposure received by patients for a pair of images; and
- linearity, uniformity, and repeatability of the system.

The results of the evaluation could be applied for optimal daily use of the system, as benchmark data in a quality assurance (QA) procedure, and to compare with other similar IGRT systems.

## II. MATERIALS AND METHODS

### A. Novalis image‐guided system

**Figure 1:**
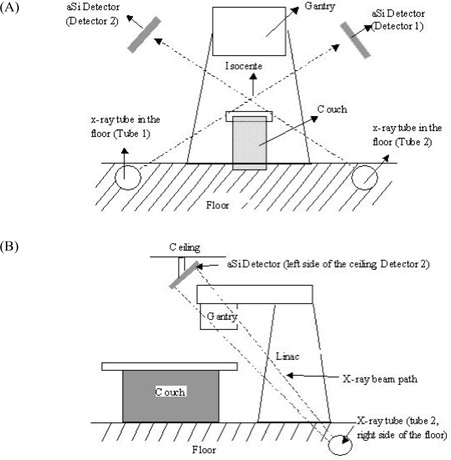
Schematic of the two X‐ray systems in radiosurgery treatment room. (A) Frontal view. (B) Side view.

The Novalis IGRT system consists primarily of two floor‐mounted kilovoltage X‐ray tubes that project obliquely from lateral to medial, posterior to anterior, and superior to inferior onto two corresponding flat‐panel detectors mounted on the ceiling (Fig. 1), and an infrared external marker monitoring subsystem. The objectives of the infrared subsystem are twofold:

- to perform the initial patient setup according to external skin markers, and
- to control the patient and couch positions with superior accuracy once position deviation is determined by image fusion.

After initial patient setup with the infrared subsystem, two X‐ray images from the pair of flat‐panel detectors are acquired and fused with the 3D CT simulation images. The auto‐fusion software provides fusion algorithms with either three degrees of freedom or six degrees of freedom, called “3D fusion” and “6D fusion” respectively.

The 3D fusion technique fuses two 2D localization X‐ray images with two corresponding digitally reconstructed radiographs (DRRs) from the CT images with fixed angles. The process is formally known as 2D/2D image registration. The 6D fusion technique fuses 2D localization X‐ray images directly with the 3D CT simulation images. Specifically, the software compares the two X‐ray images with corresponding DRRs calculated from the CT images with various rotational shifts and finds the pair of DRRs with maximal similarity to the X‐ray images to be the best match. This 6D fusion is formally known as 2D/3D image registration. A phantom study demonstrated that, as compared with 3D fusion, 6D fusion improves localization accuracy when rotational deviation is present.^(12)^

### B. Overall performance of the Novalis IGRT system

Image fusion, accurate position control, and coincidence between the IGRT and LINAC coordinate systems are three key factors for overall performance of an IGRT system. Specifically, a daily check of the consistency of the coordinate systems should be an important QA procedure.

In the Novalis system, the X‐ray tubes and the flat panel detectors are all mounted in fixed positions (Fig. 1). This fixed, symmetric, stereo arrangement may have better geometric accuracy and ease of operation than does a movable configuration of the detector and the tube. However, the isocenter of the IGRT system is not physically fixed. Rather, it is determined by software at each daily calibration. The treatment‐room laser system is used as the reference to correlate the IGRT isocenter with the LINAC isocenter.

This isocenter correlation requires a two‐step procedure. The first step is to use an isocenter calibration phantom to calibrate the isocenter of the infrared subsystem with the isocenter of the LINAC. The isocenter calibration phantom has 5 fixed infrared markers and an isocenter defined by 3 orthogonal lines. The room laser system is used to position the phantom in the LINAC isocenter. Two infrared cameras read the positions of the markers and hence calibrate the coordinates of the infrared system to those of the LINAC according to the known geometric relationship between the infrared markers and the isocenter in the phantom. In the second step, an X‐ray calibration phantom with multiple external infrared makers and internal radiopaque markers is used to calibrate the isocenter of the X‐ray system to the isocenter of the infrared subsystem. The infrared external markers and cameras are used to align the phantom to the isocenter of the infrared subsystem. Two radiographic images are then acquired, and the positions of the radiopaque markers are automatically identified. The isocenter of the X‐ray system is hence calibrated according to the known geometric relationships.

A Rando phantom implanted with a ball bearing (BB) 2 mm in diameter was used to evaluate the performance of the IGRT system and to verify isocenter coincidence between the IGRT and LINAC systems. The phantom was scanned using an AcQSim CT (Philips Medical Systems, Andover, MA) simulator set to a slice thickness of 3 mm. The CT images were then imported into the BrainScan treatment planning system, and a simple plan was created with the isocenter at the center of the BB. The plan and corresponding CT images were transferred to the IGRT control system.

Next, the phantom was set up with an intentional shift of about 1 cm in an arbitrary direction from the isocenter. Two corresponding localization X‐ray images were acquired, and a 6D fusion was performed to compare the X‐ray images with the CT images. The implanted BB was excluded from the image during the 6D fusion. The 6D fusion provided both translational and rotational position deviations of the phantom, although only the translational correction was made to move the phantom to the correct isocenter position. Two verification X‐ray images were acquired to confirm that the center of the BB was consistent with the isocenter of the X‐ray images. Anterio–posterior and lateral megavoltage portal films were also taken to check consistency with the LINAC isocenter.

### C. Image quality of the system

The image quality of an IGRT system is determined mainly by spatial resolution and contrast resolution. The spatial resolution is related to the focal spot size of the X‐ray source, the pixel size of the flat‐panel detectors, and the configuration of the system. The two X‐ray tubes in the system each have two focal modes corresponding to focal spots of 0.6 mm and 1.2 mm (Varian R‐21: Varian Medical Systems, Palo Alto, CA). The flat‐panel detectors have a pixel size of 0.4 mm with a sensitive area of 20.5×20.5 cm (model XRD 512–400 AL1: PerkinElmer, Waltham, MA). The spatial resolution can be quantified by the modulation transfer function (MTF) of the system. The contrast resolution is determined by many factors, such as the initial subject contrast, the X‐ray quality, the scattering radiation, and the number of photons used to take the images. Unlike the contrast in a film system, the contrast of the digital image is affected mainly by the signal‐to‐noise ratio.

**Figure 2:**
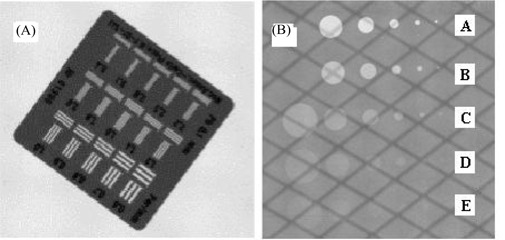
Test tools used for image resolution analysis. (A) X‐Ray image acquired for the line pair phantom (140 kVp, 50 mA, 10 ms). Line pairs are 0.6, 0.7, 0.8, 0.9, 1.0, 1.2, 1.4, 1.6, 1.8, 2.0, 2.2, 2.5, 2.8, 3.1, and 3.4 per millimeter. (B) X‐Ray images of the Las Vegas phantom (140 kVp, 100 mA, 63 ms).

To measure the MTF, we used a line‐pair template made of Plexiglas 2 mm thick with embedded lead (0.1 mm thick). The template contains a total of 15 line pairs with spatial frequencies ranging from 0.6 lp/mm to 2.2 lp/mm [see Fig. 2(A)]. We used the relative MTF (RMTF)

(1) $$RMTF=\frac{MTF(f)}{MTF(f_{1})}$$

to quantify the spatial resolution.^(^ ^13^ ^–^ ^15^ ^)^ The RMTF is the ratio of the MTF at a specific frequency to the MTF at the lowest frequency of a line‐pair template. In the present study, the 0.6 lp/mm line pair, the lowest frequency in the template, was used as the normalization—that is, $f_{1}=0.6$ lp/mm. To acquire more accurate RMTF values, the horizontal and vertical pairs of the same frequency were both taken for a region of interest (ROI) for both detector panels. The critical frequency, $f_{50}$ (the point at which the response frequency is 50% of the maximum RMTF) can also be determined from the MTF plots.

An aluminum Las Vegas phantom [Fig. 2(B)] was used to check the contrast resolution of the image system. This phantom has a matrix of 5×6 circular holes of various diameters and depths [Fig. 2(B)]. Contrast and spatial resolution could both be measured in this phantom.

The Las Vegas phantom was set at the isocenter visually parallel to the flat panel. Images were taken using various kilovoltage and milliampere second settings. The contrast and contrast‐to‐noise ratio (CNR) can be calculated from

(2) $$Contrast=\frac{\left(X_{out}-X_{in}\right)}{X_{out}}\;\text{and}\;CNR=\frac{\left(X_{out}-X_{in}\right)}{\sigma },$$

where $X_{\text{out}}$ and $X_{\text{in}}$ are, respectively, the average pixel value outside and inside the hole and σ is the standard deviation of the pixel number inside the hole.

### D. X‐ray exposure measurement

A parallel‐plate ion chamber (Model 96035, 15 cm^3^ sensitive volume size: Keithley Instruments, Cleveland, OH) was used to estimate entrance dose to the patient by exposure measurement. The exposure was measured at the isocenter. The chamber's surface was set parallel to the detector panel with sufficient back‐scattering buildup. The kilovoltage photons used in the measurement were varied from 40 kVp to 150 kVp. Operating current for each voltage ranged from 63 mA to 200 mA. Exposure time varied from 63 ms to 200 ms for each kilovoltage–milliampere combination. The exposures were taken for each X‐ray tube and detector set, and the values were averaged. Collected charges (in nanocoulombs) were converted to exposure using the calibration data provided by an accredited dosimetry calibration laboratory.

### E. Linearity, uniformity, and repeatability of the system

#### E.1 X‐Ray output

To quantify the linearity and repeatability of the X‐ray output, unit exposure (mR) per unit milliampere second (that is, mR/mAs) was calculated for a fixed milliampere second at six different kilovoltage photon settings (40, 60, 80, 100, 120, and 150 kVp).^(13)^ In the present study, 16 mAs was used with four different milliampere and millisecond combinations (that is, 80 mA×200 ms, 100 mA×160 ms, 160 mA×100 ms, and 200 mA×80 ms). The linearity of the output was then determined by

(3) $$\%Linearity=\frac{\left(\frac{Maximum\;mR/mAs-Minimum\;mR/mAs}{2}\right)}{Average\;mR/mAs}\times 100{\%}^{,}$$

where Maximum mR/mAs, Minimum mR/mAs, and Average mR/mAs are, respectively, the largest, the smallest, and the average of the four measurements.

The repeatability of the X‐ray output was calculated^(13)^ using

(4) $$\%\;\mathrm{Repeatability}=\frac{\left(\frac{Maximum\;mR-Minimum\;mR}{2}\right)}{Average\;mR}\times 100{\%}^{,}$$

where Maximum mR, Minimum mR, and Average mR are, respectively, the largest, the smallest, and the average of the four consecutive measurements. According to Gray et al.,^(16)^ repeatability should be maintained within ±5% for the diagnostic X‐ray system.

#### E.2 Flat‐panel detectors

To measure linearity and uniformity of the flat‐panel detectors correctly, a gain and offset calibration was performed before the measurements. This calibration corrects for the defected pixels and the background pattern of the X‐ray tube cover by adjusting the gain and offset to the images taken in air.

“Linearity” measures the relationship of X‐ray output to pixel reading within the linear dynamic range. A ROI of 50×50 pixels was defined at five different locations on the image, and pixel values were averaged. The measurements were made at 100 kVp and 120 kVp for both detectors without using any object. Current was set to 63 mA, and time was varied from 63 ms to 160 ms, with 1 mm of Cu added to the X‐ray beam path to prevent early saturation of the image.

“Uniformity” measures how uniform the pixel reading is inside a ROI under uniform exposure. “Repeatability” checks the stability of the pixel reading when no object is under exposure.^(13)^ To avoid saturation of the flat panel detectors in the present study, uniformity and repeatability were tested at 63 kVp and 80 kVp with 63 mA and 63 ms, instead of the higher kilovoltage and milliampere settings used in the daily clinical setting. Six images were acquired on 6 different days using the same image settings. The central regions of the images obtained (100×100 pixels) were processed.

## III. RESULTS

### A. Overall performance of the Novalis IGRT system

**Figure 3:**
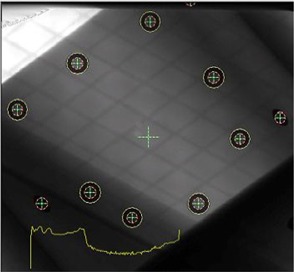
An X‐ray calibration phantom for daily isocenter calibration to ensure that the isocenter of the image‐guided radiation therapy system is consistent with the laser system. The system reads the position of each radiopaque marker and determines the isocenter position of the X‐ray system.

Fig. 3 shows a kilovoltage X‐ray image of the calibration phantom with multiple radiopaque markers. The phantom was aligned with the isocenter of the infrared subsystem, which was calibrated to the LINAC isocenter according to room lasers. The IGRT system automatically identifies these markers and defines the isocenter position (the cross at the center of image) according to the known geometric relationship between the isocenter and the markers in the phantom. The system also warns if the isocenter position differs from the previous saved position by a value larger than the threshold value (set to 0.5 mm or 1 mm). This daily calibration procedure ensures that the isocenters of the infrared subsystem, the kilovoltage X‐ray system, and the LINAC system coincide.

**Figure 4:**
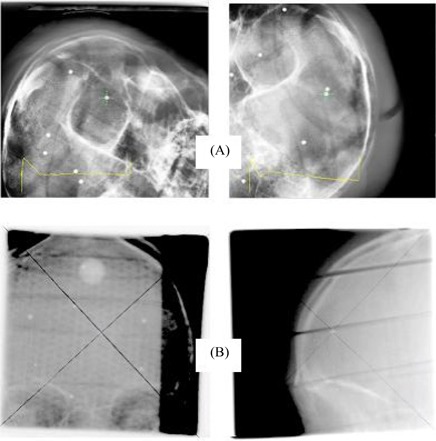
The six‐dimensional auto‐registration software can achieve submillimeter localization accuracy. (A) The image‐guided radiation therapy (IGRT) isocenter coincides with the treatment planning isocenter selected at the center of the ball bearing after auto‐registration. (B) The IGRT isocenter coincides with isocenter of the linear accelerator, as demonstrated by the anterior–posterior and lateral megavoltage portal films.

Fig. 4(A) shows a pair of verification radiographs of the Rando phantom with the isocenter at the center of the 2‐mm BB implanted in the phantom. The images were taken after the phantom's position was re‐adjusted according to the 6D fusion result. As shown in the amplified image in the right upper corner, the isocenter of the X‐ray system represented by the cross coincides with the center of the BB very well. This demonstration provides an example of excellent performance of automatic image fusion and position readjustment in the Novalis system.

Fig. 4(B) shows the anterior–posterior and lateral megavoltage portal films taken at the same position. The isocenter of the LINAC was determined by the intercepting point of two diagonal lines of the 9.8×9.8‐cm field. As shown in the amplified images at the left upper corner of the films, the LINAC isocenter coincides with the center of the BB very well. This demonstration shows that the isocenter of the X‐ray system correlates well with the isocenter of the LINAC. A comprehensive study of the performance of 3D fusion was presented by Yan et al.^(17)^ A comparison of the system's 3D fusion with its 6D fusion was reported by Jin et al.,^(12)^ and a comprehensive evaluation of the 6D fusion was also performed by Jin and colleagues.^(18)^

### B. Image quality

#### B.1 Spatial resolution

Fig. 2(A) shows a typical image of the line pair tool taped on the surface of the flat panel detector, acquired with an X‐ray technique of 140 kVp, 50 mA, and 10 ms, with a focal spot of 1.2 mm. The average pixel value and the standard deviation of a ROI including just a line pair were obtained, and the MTF for each line pair was calculated.

**Figure 5:**
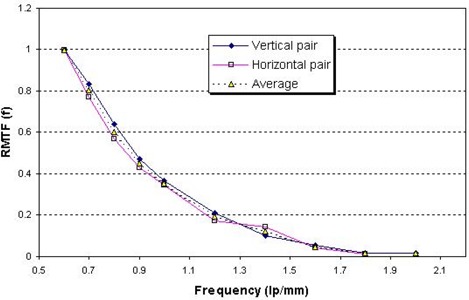
The relative modulation transfer function (RMTF) curves used at 140 kVp, 50 mA, and 10 ms.

Fig. 5 shows the RMTF curves for flat‐panel detector 1 at 140 kVp from both the vertical and horizontal line patterns and the average of the two. Using interpolation and curve‐fitting, we obtained $f_{50}$, the RMTF at critical frequency (point where response frequency is 50% of the maximum RMTF). The average $f_{50}$ for flat panel detector 1 was 0.87 lp/mm. The discrepancy of $f_{50}$ between the vertical and horizontal line patterns was less than 10%. We found that the RMTF varied little with various kilovoltage photon and milliampere second settings in the general working range. However, the RMTF for flat panel detector 2 seemed to be slightly different from that of detector 1, with the $f_{50}$ for detector 2 being 0.7 lp/mm.

The RMTF of an imaging device depends on the configuration of the X‐ray source and the flat‐panel detector, in addition to the characteristics of source and the detector. Considering that the two X‐ray tubes and the two flat‐panel detectors were identical in terms of their manufacture and model, and that the two imaging devices have a symmetric configuration, our finding suggests that variations exist for the same model of these devices. An acceptance test would helpful to ensure a high standard for the devices when they are installed. Nevertheless, the measured $f_{50}$ can be used as a benchmark for checking any degradation of the system and for annual QA of the devices.

#### B.2 Contrast resolution

**Figure 6:**
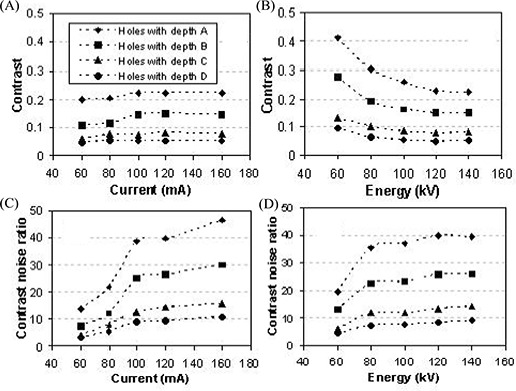
Measured contrast and contrast‐to‐noise ratio (CNR). (A) Contrast compared with milliamperes. (B) Contrast compared with kilovoltage photon. (C) CNR compared with milliamperes. (D) CNR compared with kilovoltage photon. For the foregoing measurements, kilovoltage photon was 140 kV for (A) and (C), milliamperes was 120 mA for (B) and (D), and milliseconds was 100 ms for all measurements with the exception of one data point, which was 63 ms when milliamperes was 160 mA and kilovoltage photon was 40 kV.

Fig. 2(B) shows a typical image of the Las Vegas phantom supported on the treatment table with the phantom surface parallel to the flat‐panel detector. The X‐ray technique was 140 kVp, 100 mA, and 63 ms. The image of the tennis racquet can clearly be seen. For measurement of the pixel value inside a hole, only a small area of ROI excluding the tennis racquet pattern was measured. Only holes with depths A, B, C, and D were measured. Fig. 6(A,B) shows the contrast with varying milliamperes and kilovoltage photons used for the X‐ray images for four different sets of holes at varying depths. For the same holes, Fig. 6(C,D) shows the CNR varying with the milliamperes and kilovoltage photons used for the X‐ray images. For those measurements, the kilovoltage photon setting was 140 kV for various milliamperes [Fig. 6(A,C)], and the milliampere setting was 120 mA for various kilovoltage photons [Fig. 6(B,D)]. The millisecond setting was 100 ms for all the measurements with the exception of one data point, which was 63 ms when the milliampere and kilovoltage photon settings were 160 and 140 respectively.

We noted that

- contrast increased with milliamperes from 60 mA to 100 mA, tending to saturate above 100 mA.
- contrast decreased with the kilovoltage photon setting.
- CNR increased rapidly with milliamperes from 60 mA to 100 mA and slowly with milliamperes from 100 mA to 160 mA.
- CNR increased with the kilovoltage photon setting.

The CNR usually correlates with the number of quanta received by the detectors, which is proportional to the milliampere seconds. That situation is consistent with our finding that the contrast increased with the milliampere seconds. We found that, for a CNR of approximately 4, the smaller holes were difficult to identify with the human eye.

### C. Exposure measurement

**Figure 7:**
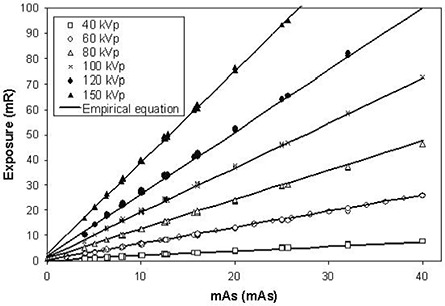
Exposures plotted at various kilovoltage photon and milliamperes, compared with the empirical equation.

Fig. 7 shows the average exposure (mR) plotted against milliampere seconds at various kilovoltage photon settings. The highest exposure was about 95.1 mR (0.0826 cGy in air) at 150 kVp, 160 mA, and 160 ms, the maximum allowable setting of the X‐ray tube. We noted that the average exposures correlated linearly with the multiplication of beam current (mA) and exposure time (s)—namely, the milliampere seconds. As shown in Fig. 7, the correlation of average exposure with the kilovoltage photons and the milliampere seconds can be expressed in the empirical equation

(5) $$Exposure=\left[0.949\times {\left(\frac{kVp}{100}\right)}^{2}+1.33\times \left(\frac{kVp}{100}\right)-0.502\right]\times (mAs+0.75),$$

where Exposure is expressed in mR; kVp, in kilovoltage; and mAs in milliampere seconds. We found that tube 1 had a slightly different exposure than that of tube 2 at the same nominal setting (<6%). Below 100 kVp, tube 1 showed a higher exposure than did tube 2. The situation was reversed beyond 125 kVp. Given the same kilovoltage photons, the discrepancy was more prominent when low milliamperes were supplied.

### D. Linearity and repeatability of the system

#### D.1 X‐Ray output

As shown in Fig. 7, the X‐ray output (in terms of exposure) correlated linearly with the milliampere seconds very well. Quantitative data can be extracted by analyzing four settings of measurements with the same milliampere seconds (80 mA×200 ms, 100 mA×160 ms, 160 mA×100 ms, and 200 mA×80 ms). Using equation 3, the linearity was calculated to be less than 4% for all kilovoltage photon settings. This result is well within the ±10% limit recommended by the American Association of Physicists in Medicine.^(19)^ The same four sets of measurements were used for repeatability. At a very low kilovoltage photon setting (40 kV), we found that the result was less satisfactory and that repeatability calculated by equation 4 was 10.2%. However, the values were well within 5% from 50 kVp to 100 kVp, and were less than 1.5% beyond 100 kVp (the common range in the clinical setting). The X‐ray output was checked 1 year later and showed a consistency of better than 3%.

#### D.2 The flat‐panel detectors

Evaluation of uniformity, linearity, and repeatability of the detector panels was performed after a gain‐and‐offset calibration procedure. This calibration is usually performed annually, whenever a change of configuration occurs, or whenever image quality degradation is suspected. The uniformity was obtained by comparing 5 different ROIs across the white images obtained (images acquired with nothing between the X‐ray tube and the detector except for the floor cover). Percentage deviations of uniformities were 0.72% – 1.27% and 1.6% – 2.04% for detectors 1 and 2 respectively.

**Figure 8:**
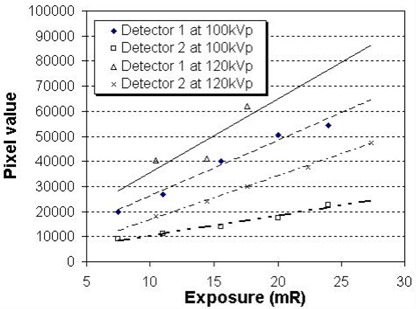
Linearity for detector 1 and detector 2 at 100 kVp and 120 kV.

**Figure 9:**
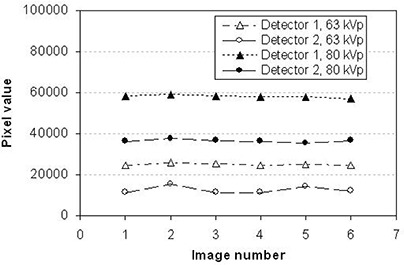
Repeatability of pixel value of the flat panel detectors for 6 different days at 2 different kilovoltage photon settings.

Figs. 8 and 9 respectively show the linearity and repeatability for the two detectors at two different technical settings. Detector 2 had excellent linearity and detector 1 had reasonable repeatability for both settings. However, detector 1 showed relatively poor linearity at a higher kilovoltage photon setting (120 kV), and detector 2 showed reading variations on different days at a lower kilovoltage photon setting (63 kV). In addition, the pixel value of detector 1 was about 1.6 – 2.5 times higher than that of detector 2 at the same exposure condition. These findings could be the result of

- different calibration gain and offset for the two detectors, and
- a limited time range of validity for the calibration linearity and stability.

Further investigation revealed that detector 2 was replaced after about 1 year of clinical use with a new detector of the same model. In addition, the investigation found that synchronization between the time of X‐ray exposure and the time of integrating the charges in each pixel of the detector might be unstable because of unknown time delays in the control system. After the synchronization control hardware was upgraded, the linearity and repeatability of both detectors in varying exposure conditions were checked and found to be improved.

## IV. DISCUSSION

We evaluated the performance of a Novalis IGRT system and the characteristics of its kilovoltage X‐ray imaging device. As demonstrated in Fig. 4(A), the IGRT system is able to localize an isocenter position with an excellent accuracy even when the initial setup position has a large position error. Reliable image fusion and accurate patient position control are key factors in achieving accurate isocenter localization. The ExacTrac subsystem (infrared markers) can adjust the position of the treatment table with a precision better than 0.3 mm.^(20)^ High quality of the localization and simulation images is a necessity for reliable image fusion. We measured an overall RMTF of the imaging device of about 0.7 – 0.9 lp/mm at $f_{50}$ and about 1.0 lp/mm at $f_{30}$. That finding suggests that 1‐mm objects can be fairly resolved with the kilovoltage X‐ray imaging device. The X‐ray images also showed superior contrast resolution in optimal exposure conditions. In addition, the unique configuration of the X‐ray system should also be credited for the superior performance. A fixed configuration of X‐ray tubes and flat‐panel detectors can eliminate any potential spatial uncertainties caused by mechanical movement. A large source‐to‐detector distance reduces the solid angle of the radiation beam and hence reduces potential geometric distortion. A large isocenter‐to‐detector distance reduces the potential body scattering to the detectors and hence increases the CNR.

A reasonable CNR for the imaging device is essential for reliable image fusion. Our clinical experience indicates that poor contrast of the localization images is usually associated with unreliable image fusion. The CNR tended to increase with the kilovoltage photon setting and the milliamperes. However, it should be pointed out that the CNR measured in this study entirely lacked attenuation, and X‐ray attenuation attributable to a large body could greatly reduce the CNR.

The Novalis IGRT system has a unique configuration of oblique beam entrance. The total beam path length (*L*) in a patient's body will be larger than the diameter (*D*) of the patient. The path length *L* can be expressed as L=D/(sin γ), where γ≈35 degrees is the oblique entrance angle.^(18)^ Therefore, for a large patient with a diameter of 35 cm, the total radiation path length can be as large as 61 cm. This path length greatly degrades the contrast resolution of the kilovoltage X‐ray image, and hence the image quality, and finally the localization accuracy and reliability. The maximum kilovoltage photon and milliampere settings should be used for large patients. Powerful X‐ray tubes with larger kilovoltage photon and milliampere capabilities could be helpful in improving the image quality in such applications.

The coincidence of the coordinates of the IGRT and LINAC systems is another key factor in ensuring accurate targeting. The Novalis system uses a daily two‐step calibration procedure to ensure that the infrared subsystem, the kilovoltage X‐ray system, and the LINAC system have a consistent isocenter. However, the calibration procedure is based on the room lasers as the reference for the LINAC isocenter. The room lasers could demonstrate small shifts for various reasons or could even be accidentally adjusted by an unauthorized individual. A daily or weekly verification of the accuracy of the room laser system should be an essential part of QA. This verification could use the test demonstrated in Fig. 4. A Winston–Lutz test could also serve the purpose.

The spatial resolution of the simulation CT images could be another factor affecting the reliability of image fusion and subsequently target localization accuracy. The simulation CT images usually have an excellent spatial resolution in the axial direction and a relatively poor resolution in the longitudinal direction, depending on the slice thickness used in CT scanning. The CT slice thickness could affect target localization accuracy in two ways:

- Uncertainty of target definition in the CT images (such as locating the center of a BB)
- Uncertainty of image fusion

Martin Murphy has noted that the CT slice thickness can be a dominant factor for the uncertainty of image fusion when the edge definition in the DRR is as sharp as in the actual radiograph.^(21)^ His simulation study showed that the distribution of image fusion errors (the root mean square) declined by a factor of 2 (from 0.4 mm to 0.2 mm) when CT slice thickness was reduced from 3 mm to 1.5 mm. Yan et al. also showed that the CT slice thickness affected the accuracy of target localization with 3D fusion,^(17)^ although the difference in localization accuracy between 2‐mm and 3‐mm CT slice thickness was relatively small. Those results suggest that improving the spatial resolution of both the localization and the simulation images can improve fusion accuracy and subsequently overall localization accuracy. However, it was recently noted that no significant difference in overall targeting accuracy occurred between 2‐mm and 3‐mm CT slice thicknesses when 6D fusion was used.^(12)^ In that study, LINAC portal films were used to determine the targeting accuracy. Because many other factors—such as the uncertainty of LINAC isocenter position (the isocenter could move as the gantry rotates) and the uncertainty in the calibration of the isocenters of the kilovoltage X‐ray and the LINAC—could also contribute to the overall uncertainty of targeting, the foregoing findings suggest that the improvement in image fusion accuracy attributable to the reduction of CT slice thickness from 3 mm to 2 mm is relatively small as compared with other factors.

An empirical equation for estimating patient exposure with different technical parameters has been derived. The maximum entrance dose (skin dose) delivered to patients is less than 0.083×2∼0.2 cGy for a pair of X‐ray images. That dose is negligible compared with the radiation dose delivered to the target, especially for radiosurgery cases. Multiple images can be taken to monitor patient movement and to readjust patient position between fields if such movement occurs. However, for applications involving continuous tracking of patient movement, such as for respiratory motion tracking or gating using an internal surrogate,^(22)^ images have to be taken at a much higher frequency (>10/s) to reduce potential time delays.^(23)^ The total dose could be significant, and optimal technical settings should be used in such an application.

The basic characteristics of the image device—for example, RMTF, contrast resolution, CNR, exposure, uniformity, and linearity—have been established for the Novalis IGRT system. Most of these parameters might be stable during the normal operational lifetime of the system. However, the target of the X‐ray tube could gradually be worn out, the flat‐panel detector could be degraded, the configuration of the system could be modified unexpectedly for various reasons, and the synchronization between the times of X‐ray exposure and detector reading could fail because of unexpected problems in the control system. It is therefore recommended to take such measurements during the acceptance test or commissioning of the system to establish benchmark data. These data can then be used as the references or standards for a regular annual QA check or periodic checks because of degradation in image quality or reliability of the system. On the other hand, because the dose from a pair of localization images to the patient is negligible as compared with the treatment dose, standards related to patient exposure, such as the linearity and repeatability of the IGRT system, could be much looser than their counterparts in radiology. Inputs from various users of the system and from other different IGRT systems are needed to establish such standards.

## V. CONCLUSION

We systematically evaluated the characteristics of the Novalis X‐ray IGRT system. The results indicate that the Novalis IGRT system is a safe and reliable target localization device suitable for clinical use. The measured data could be used for optimizing the daily use of the system and helping to establish benchmark data for QA procedures.

## ACKNOWLEDGMENTS

The authors acknowledge Mr. Stephan Erbel and Mr. Armin Fuerst in BrainLab, Germany, for technical advice provided.

## Supporting information

Supplementary Material
