# Supplementary figures and images for: Clinical assessment and characterization of a dual‐tube kilovoltage X‐ray localization system in the radiotherapy treatment room
Source: J Appl Clin Med Phys. 2008 Jan 13;9(1):1–15. doi: 10.1120/jacmp.v9i1.2318 (PMC5721528; doi:10.1120/jacmp.v9i1.2318)

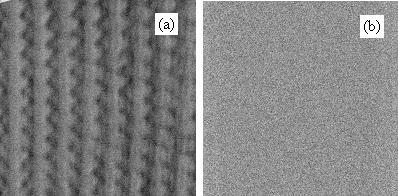

Supplement: Supplementary file 1 — Supplementary Material [file ACM2-9-01-s001.jpg]
